# Transcriptome and DNA methylome analyses reveal underlying mechanisms for the racial disparity in uterine fibroids

**DOI:** 10.1172/jci.insight.160274

**Published:** 2022-10-24

**Authors:** Emmanuel N. Paul, Joshua A. Grey, Tyler J. Carpenter, Zachary B. Madaj, Kin H. Lau, Scott A. Givan, Gregory W. Burns, Ronald L. Chandler, Ganesa R. Wegienka, Hui Shen, Jose M. Teixeira

**Affiliations:** 1 Department of Obstetrics, Gynecology and Reproductive Biology, College of Human Medicine, Michigan State University, Grand Rapids, Michigan, USA.; 2 Bioinformatics and Biostatistics, Van Andel Institute, Grand Rapids, Michigan, USA.; 3 Department of Public Health Sciences, Henry Ford Hospital, Detroit, Michigan, USA.; 4 Department of Epigenetics, Van Andel Institute, Grand Rapids, Michigan, USA.

**Keywords:** Reproductive Biology, Epigenetics, Molecular genetics, Obstetrics/gynecology

## Abstract

Uterine fibroids (leiomyomas) affect Black women disproportionately compared with women of other races and ethnicities in terms of prevalence, incidence, and severity of symptoms. The causes of this racial disparity are essentially unknown. We hypothesized that myometria of Black women are more susceptible to developing fibroids, and we examined the transcriptomic and DNA methylation profiles of myometria and fibroids from Black and White women for comparison. Myometrial samples cluster by race in both their transcriptome and DNA methylation profiles, whereas fibroid samples only cluster by race in the latter. More differentially expressed genes (DEGs) were detected in the Black and White myometrial sample comparison than in the fibroid comparison. Leiomyoma gene set expression analysis identified 4 clusters of DEGs, including a cluster of 24 genes with higher expression in myometrial samples from Black women. One of the DEGs in this group, von Willibrands factor (*VWF*), was significantly hypomethylated in both myometrial samples from Black women and in all fibroids at 2 CpG probes that are near a putative enhancer site and that are correlated with *VWF* expression levels. These results suggest that the molecular basis for the disparity in fibroid disease between Black and White women could be found in the myometria before fibroid development and not in the fibroids themselves.

## Graphical abstract

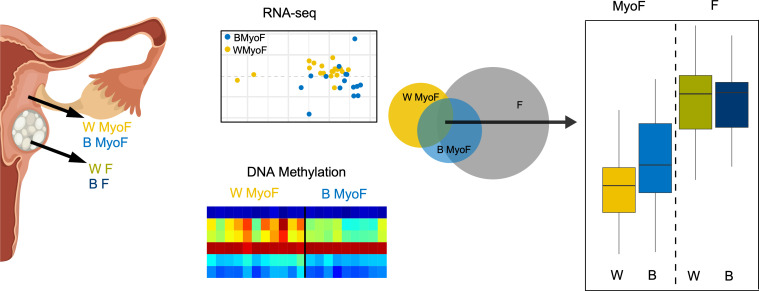

## Introduction

Uterine fibroids, also known as uterine leiomyomas, are the most common tumors in women during their reproductive years, with an incidence of up to 80%, depending on race or ethnicity (1–3). Although benign, fibroids can significantly impact the quality of life for many women, with approximately 25% of women with fibroids seeking clinical care for their symptoms (4, 5). There is a strong racial disparity in fibroid disease incidence and severity; Black women are 2–3 times more likely to develop clinically significant fibroids (1, 6, 7), to have them at an earlier age (8, 9), and to report more and worse fibroid symptoms (most commonly, pain and heavy menstrual bleeding) (10).

Hysterectomy, which ends a woman’s fertility, is the only definitive treatment available for the disease, and fibroids are the most common indication for hysterectomy in the United States (11). Short-term therapies, including Lupron, selective progesterone receptor modulators (SPRMs), and oral gonadotropin-releasing hormone (GnRH) agonists (12–14), are available and can provide some relief for women with symptomatic fibroids, but their long-term use is mostly proscribed because of associated side-effects (15). The current gap in knowledge about the sources of racial disparity in incidence and severity of fibroids limits options for prevention or treatment. These limitations leave Black women with a greater healthcare burden overall, in which they are forced to make decisions about treatment at younger ages than White women — decisions that have consequences for their fertility and the need for future treatments.

The causes of these racial disparities are unknown, but a metaanalysis demonstrated that chronic psychological stress was weakly associated with the prevalence of fibroids (16–18). Some evidence suggests that vitamin D (VitD) deficiency/insufficiency, which is endemic in Black women in the United States, could be contributing to the disparity in both the incidence and severity of the disease (17, 19–21). Previous studies have identified candidate factors and genetic differences associated with the racial disparity in disease incidence that could be involved (22), including polymorphisms of genes involved in estrogen signaling (23), retinoic acid pathway–related genes (24), and miRNA expression levels (25). However, the downstream pathways and mechanisms affected by any of these have not been completely elucidated. Most published fibroid studies of disease incidence and fibroid formation have concentrated on analyzing the uterine fibroids themselves. We used a different approach for the present study and analyzed the adjacent myometrial tissue in addition to the fibroid to determine the transcriptomic and epigenomic differences between the tissues associated with race. We analyzed myometrial samples from Black and White women with fibroids by comparing their transcriptomic and DNA methylation profiles across these race groups. Here, we show evidence that myometrial samples from Black women are different from those of White women, with higher expression of some stress- and fibroid-associated genes, suggesting mechanisms by which myometria of Black women may be more susceptible to uterine fibroid development.

## Results

**Figure 1:**
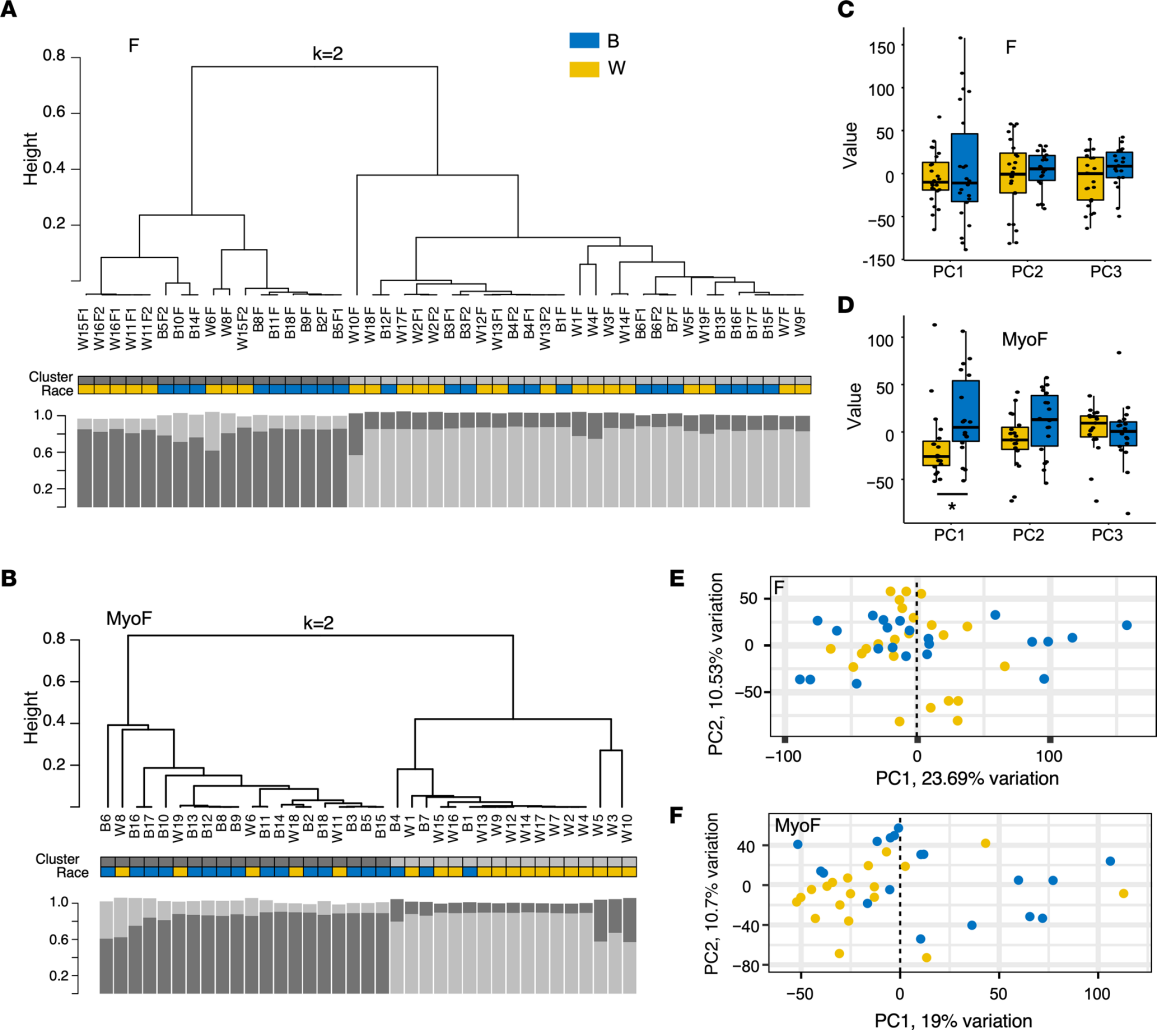
Race-based clustering of RNA-Seq results from myometrial and fibroid samples. (**A** and **B**) Whereas unsupervised hierarchical *k* = 2 means clustering of RNA-Seq results from *MED12*mt fibroid samples (*n* = 24 F White and *n* = 22 F Black) is not significantly associated with race (Fisher’s exact test, *P* = 0.76) (**A**), the results from myometrial samples (*n* = 19 MyoF White and *n* = 18 MyoF Black) show an association with race (Fisher’s exact test, *P* = 8.3 × 10^–4^) (**B**). The length of each leaf in the dendrograms indicates degree of dissimilarity. Race of each sample is color coded as indicated. The clusters are shown above the race. Spearman bootstrap analyses (1,000×) are shown below each dendrogram for each sample. The *y* axis of bootstrap columns indicates stability of clustering. (**C** and **D**) Box plots of the first 3 principal components (PC) of RNA-Seq results from Black and White F (**C**) and MyoF (**D**). The value of each sample represents the individual gene expression values transformed by the rotation matrix estimated from the PCA. Only PC1 in MyoF are significantly different (likelihood ratio test, *P = 0.03). (**E** and **F**) PC plot analyses for PC1 versus PC2 of F (**E**) and MyoF (**F**) shows samples from White and Black women in 2-dimensional space. Each dot represents 1 sample.

RNA-Seq analyses were performed on *MED12-mutant* (*MED12mt*) fibroids (F) and on matched myometrium (MyoF) samples from Black (*n* = 22) and White (*n* = 24) women (Supplemental Table 1; supplemental material available online with this article; https://doi.org/10.1172/jci.insight.160274DS1) to identify differentially expressed genes (DEGs) that could be contributing to the racial disparity in uterine fibroid disease. To minimize sample variability, only tissue samples from women with *MED12*mt fibroids, which are the most common genetic subtype of uterine fibroids (26), were used in the study. Unsupervised hierarchical clustering analysis of all expressed genes in F samples from Black and White women (Figure 1A) shows that the racial identities of the transcriptomes appear randomly scattered among the 2 main branches of the dendrogram. In contrast, when the same analysis is applied to the expressed genes in the MyoF samples from Black (*n* = 18) and White (*n* = 19) women (Figure 1B), the 2 main branches segregate comparatively well. Principal component (PC) regression analysis (Figure 1, C and D) shows that PC1 in MyoF samples is significantly associated with race, which was not the case for the F samples or for PC2 and PC3 in both the MyoF and F samples. Similarly, the PC plot of PC1 and PC2 shows a better separation by race in MyoF (Figure 1F) than in F (Figure 1E). Neither F nor MyoF samples showed significant separation by race in other component comparisons (Supplemental Figure 1, A and B).

**Figure 2:**
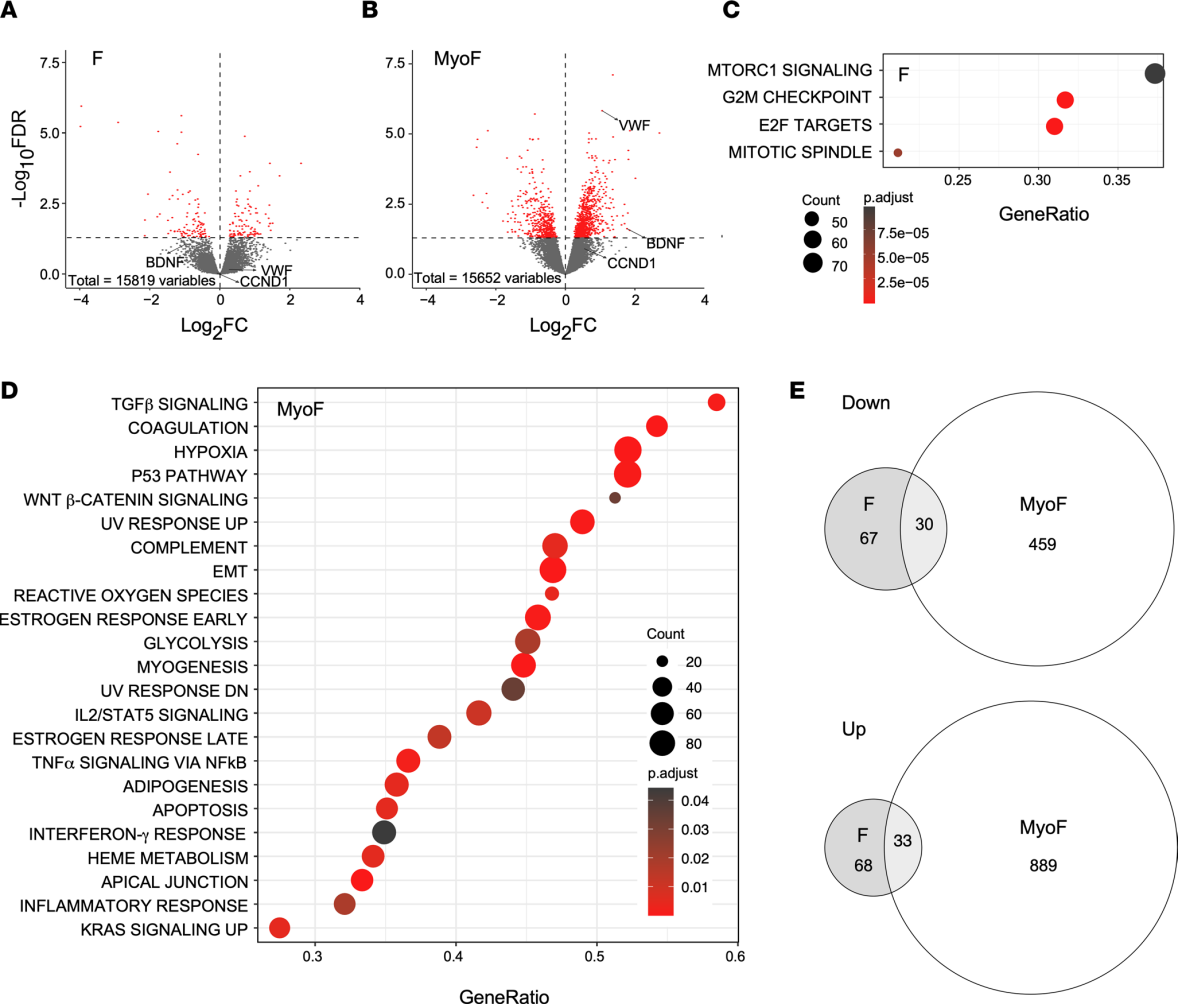
Differential gene expression by race in F and MyoF samples. Fibroids (F) from *n* = 22 Black and *n* = 24 White women, and matching myometrium (MyoF) from *n* = 18 Black and *n* = 19 White women, were compared. (**A** and **B**) Volcano plots showing the up- and downregulated genes with a FDR *P* value < 0.05 depicted as red dots in F (**A**) and MyoF (**B**). (**C** and **D**) Gene set enrichment analysis of expressed genes using Hallmark biological processes in MSigDB from the comparison of the F (**C**) or MyoF (**D**) samples from Black and White women. Gene count and significance level are shown by the size and color of each circle. (**E**) Venn diagrams illustrate the overlap of the down- and upregulated genes between Black MyoF compared with White MyoF samples and Black F compared with White F samples. Hypergeometric tests of the overlaps between the 2 comparisons of the 33 upregulated and 30 downregulated sets of overlapping genes were not significant (*P* > 0.99 for both).

Further analyses of the DEGs between transcriptomes of Black and White women revealed that more DEGs (FDR < 0.05) were observed in the MyoF samples (Figure 2B, 1,411 total DEGs; Supplemental Table 2) compared with the F samples (Figure 2A, 198 total DEGs; Supplemental Table 3). Three different genes that we thought could be differentially expressed in the tissues from Black and White women based on our previous studies (27, 28) — or that are candidate genes gleaned from the literature that could be important for stress-related fibrosis (29–33) — are highlighted. von Willebrand factor (*VWF*) and brain derived neurotrophic factor (*BDNF*) were increased in MyoF samples from Black women (referred to as Black MyoF samples) compared with MyoF samples from White women (referred to as White MyoF samples) but not in the F samples from Black women (referred to as Black F samples) compared with F samples from White women (referred to as White F samples). Cyclin D1 (*CCND1*), which we have previously reported upregulated in F compared with MyoF (27), was not significantly induced in MyoF from Black women compared with MyoF from White women. Gene Set Enrichment Analyses (GSEA) (Figure 2, C and D) was performed with Hallmark gene sets (34, 35) to identify dysregulated pathways that could be important for the racial disparity in fibroid disease. Only 4 Hallmark gene sets involved in cell growth and cell cycle were enriched in Black F compared with White F samples (Figure 2C). In contrast, 23 Hallmark pathways were enriched in Black MyoF compared with White MyoF samples (Figure 2D), including known pathways that are enriched in uterine fibroids: TGF-β signaling, Wnt/β-catenin signaling, P53 pathway, and myogenesis (36–38). Hypoxia and reactive oxygen species (ROS), stress-related pathways, were also enriched in MyoF from Black women. A total of 489 genes were downregulated and 922 genes were upregulated (Figure 2E) in MyoF samples comparing Black and White women. Only 97 genes were downregulated and 101 were upregulated in the same comparison with F samples (Figure 2E). Overlaps of the 2 comparisons were not significant.

**Figure 3:**
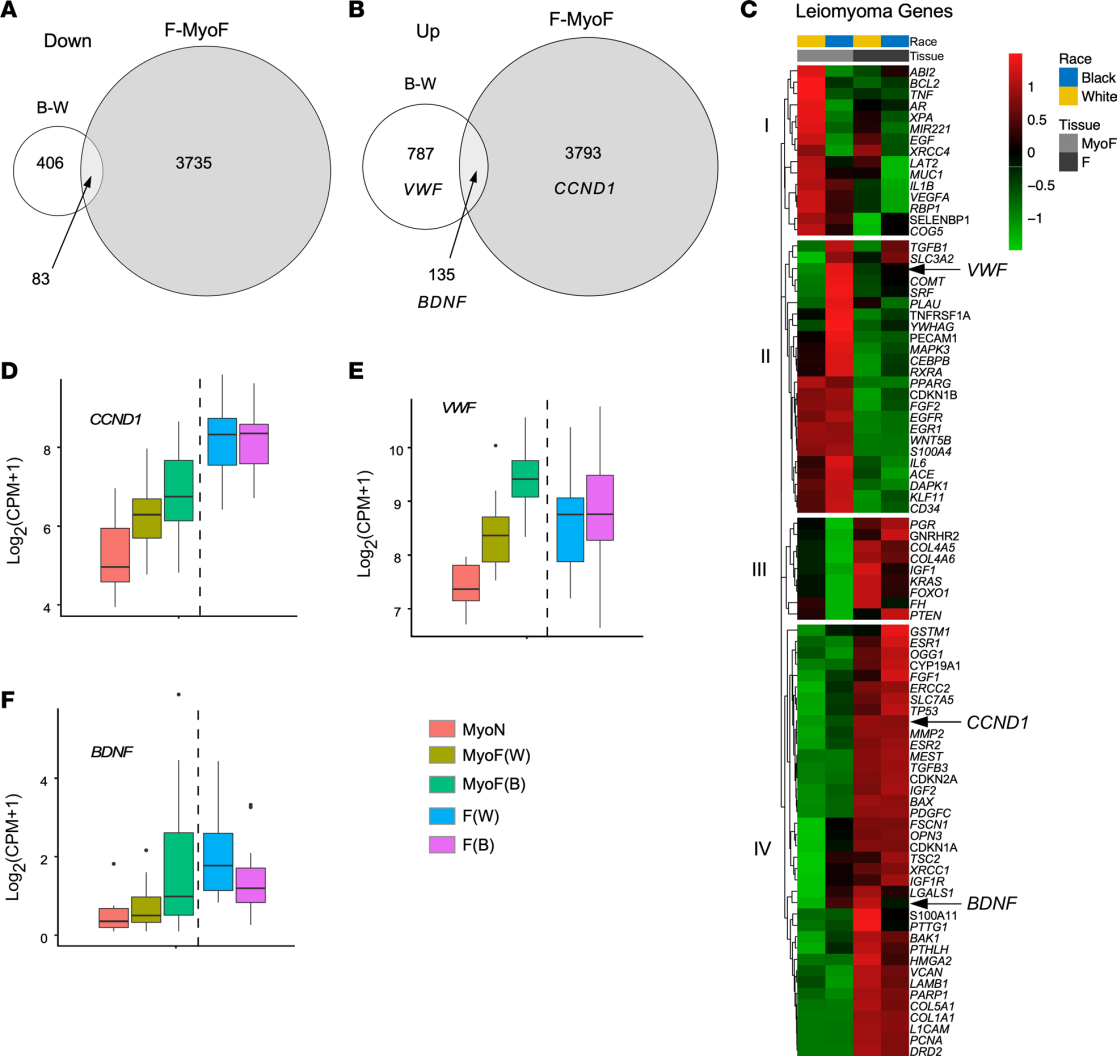
Transcriptomic similarities in fibroids (F) from all women and Black women’s matched myometria (MyoF). (**A** and **B**) Venn diagrams illustrate the overlapping downregulated (**A**) and upregulated (**B**) differentially expressed genes (DEGs) between MyoF from Black women (*n* = 18) and MyoF from White women (*n* = 19) and between F (Black and White combined, *n* = 46) and MyoF (Black and White combined, *n* = 37). Hypergeometric testing revealed that the overlaps were significant, with *P* = 9.0 × 10^–5^ for the downregulated genes and *P* = 1.9 × 10^–14^ for the upregulated genes. (**C**) Heatmap of the leiomyoma gene set from Disease Ontology with added *BDNF,* using the average log_2_(CPM + 1) of each group: MyoF White, MyoF Black, F White (each *n* = 24), and F Black (*n* = 22). Color gradient represents gene expression levels as *Z* scores. (**D**–**F**) Box plot of *CCND1* (**D**), *VWF* (**E**), and *BDNF* (**F**) of myometrium from White nonfibroid women (MyoN) (*n* = 6), MyoF from White (*n* = 19), or Black (*n* = 18) women and F from White (*n* = 24) or Black (*n* = 22) women. Gene expression is shown as log_2_(CPM + 1). FDR *P* values for each comparison are reported in the Table 1.

To determine if the DEGs in MyoF from Black women compared with those from White women contained fibroid genes, we overlapped these DEGs with the DEGs of the F versus matching MyoF comparison. More than 15% of the DEGs identified in the MyoF samples from Black women compared with those of White women were also differentially expressed in F compared with MyoF (Figure 3, A and B). Expression of *BDNF* was upregulated in the Black and White MyoF and in the F and MyoF comparisons (Figure 3B). *VWF* expression was only seen upregulated in the Black and White comparison of MyoF samples, and *CCND1* was upregulated only in F compared with MyoF (Figure 3B).

We performed a clustering analysis of a leiomyoma gene set (39) with the addition of *BDNF* for MyoF and F samples in both race groups. The analysis showed 4 distinct clusters of genes, which we have called groups I, II, III, and IV (Figure 3C). Group I contained genes with higher expression in White MyoF samples compared with Black MyoF, Black F, and White F samples. Group II included genes, such as *VWF*, that showed higher expression in the Black MyoF samples. Gene expression in group III was tissue type dependent, with low expression in all MyoF samples compared with all F samples. Lastly, group IV had highly expressed genes in F samples, such as *CCND1*, with some genes also highly expressed in Black MyoF samples, such as *BDNF*.

We next compared the gene expression profiles of the MyoF and F samples from Black and White women with the White nonfibroid myometrial (MyoN, *n* = 6) samples from a recently published study in our laboratory (27). In that study, MyoF transcriptomes appeared more like the transcriptomes of F samples than did the MyoN samples, suggesting that the myometria from women with fibroids were prone to fibroid disease or were affected by the fibroids themselves. *CCND1*, *VWF*, and *BDNF* expression levels were not significantly different between F from Black women and F from White women (Figure 3, D–F, and Table 1). *CCND1* expression was significantly higher in F tissue samples from either race compared with those from MyoF and in MyoF samples compared with MyoN. Within the MyoF samples, Black women had a higher level of *CCND1* expression than the White women (Figure 3D). *VWF* expression was significantly higher in MyoF samples (from White and Black women) compared with MyoN samples, but only the Black MyoF samples were significantly higher than F samples of either race (Figure 3E). We also confirmed that *VWF* was upregulated in Black MyoF compared with White MyoF (Supplemental Figure 1C) using data from a published study (40). *BDNF* expression was significantly increased in Black MyoF samples compared with MyoN samples and White MyoF samples. *BDNF* expression was not significantly different when Black or White F samples were compared with Black MyoF; however, *BDNF* expression was higher in F from either race compared with White MyoF.

**Figure 4:**
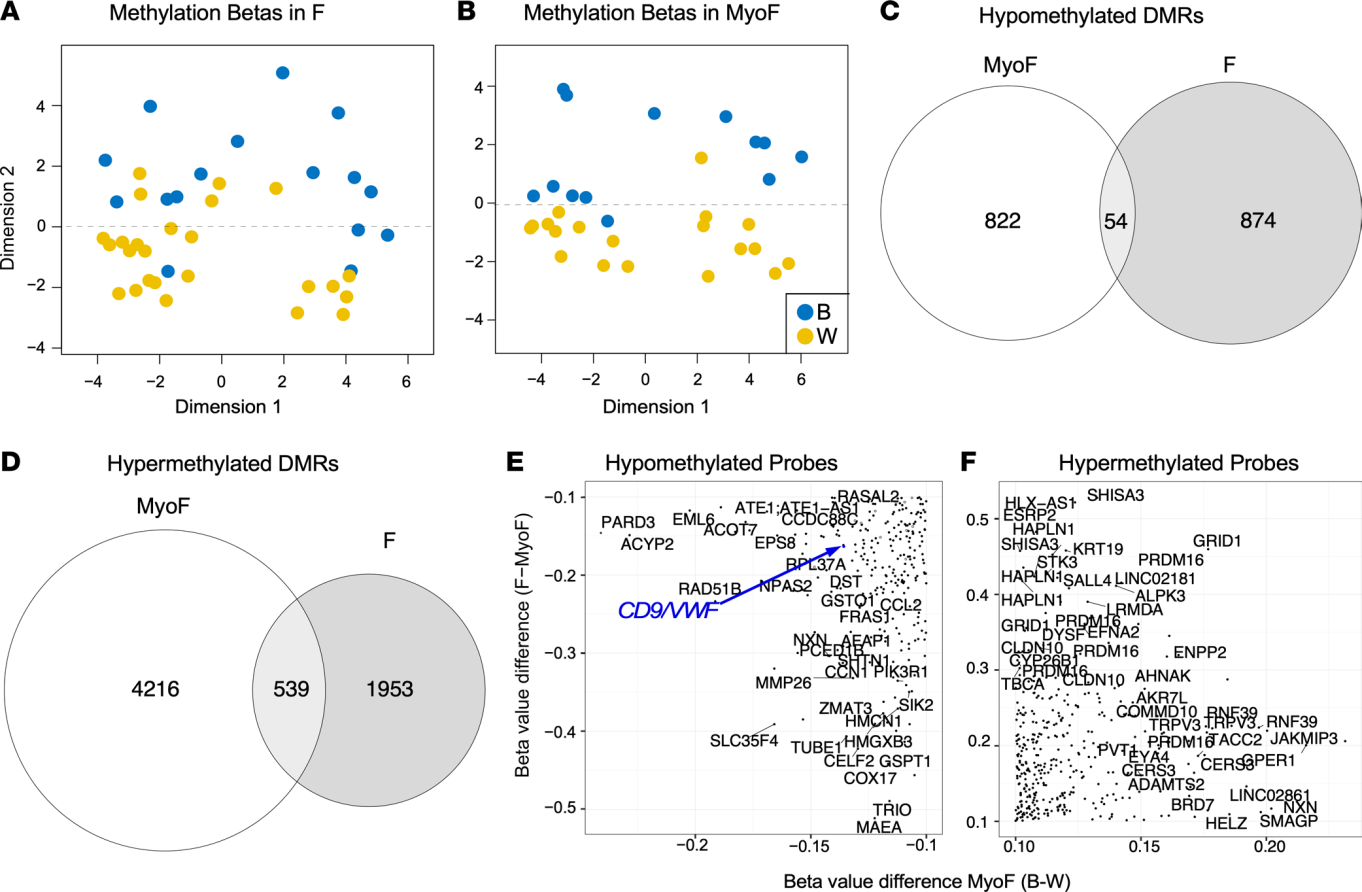
DNA methylation profiles of fibroids (F) and matched myometria (MyoF) from Black and White women. DNA methylation in the samples was determined using the Illumina MethylationEPIC Beadchip microarray. (**A** and **B**) Multidimension scaling plots of β values for fibroids (F) from Black (*n* = 16) and White (*n* = 25) women (**A**) and matching myometrial samples (MyoF) from Black (*n* = 13) and White (*n* = 19) women (**B**). Each dot represents an individual sample. Significant methylation differences by race were determined by the likelihood ratio test; *P* = 2.7 × 10^–5^ for F and *P* = 2.2 × 10^–16^ for MyoF. (**C** and **D**) Gene-associated differentially methylated regions (DMRs) for the Black and White race comparisons of MyoF and F samples are shown in a Venn diagram as hypomethylated (**C**) or hypermethylated (**D**), with the overlap in the circles indicating shared DMRs. Hypergeometric testing of the overlaps in **C** and **D** reveal that they were statistically significant (*P* = 2.6 × 10^–9^ and 2.3 × 10^–66^, respectively). (**E** and **F**) The congruent hypomethylated (**E**) and hypermethylated (**F**) EPIC CpG probes in the Black and White MyoF and the MyoF and F comparisons (>10%) are shown plotted with randomly displayed gene names. A *CD9/VWF*-associated probe is highlighted in blue.

Altered DNA methylation, both genome-wide and gene specific, has been proposed as a possible mechanism for stress-related changes observed in a variety of systems (reviewed in ref. 41). Methylomes for MyoF and F samples from Black and White women (Supplemental Table 4) were profiled using the Infinium MethylationEPIC array (EPIC) (42). Multidimensional scaling (MDS) plots based on the 500 most variable EPIC DNA methylation probes by SD revealed a significant difference in dimension 2 between Black and White women in both F (*n* = 16 Black, *n* = 25 White) and MyoF (*n* = 13 Black, 19 White) tissues (Figure 4, A and B); these differences were independent of the patient’s actual age, their inferred age, and batch effects (data not shown). Comparison of the gene-associated differentially methylated regions (DMRs) containing differentially methylated CpGs in Black and White samples (Figure 4, C and D, and Supplemental Tables 5 and 6) shows more total DMR-associate genes in the MyoF samples than in the comparison of F samples from Black and White women, reflecting the greater separation observed in Figure 4B compared with Figure 4A. The number of hypomethylated DMR-associated genes was slightly greater in F than in the MyoF samples from Black women, but the opposite was true for hypermethylated DMRs with MyoF samples showing greater numbers of gene-associated DMRs in Black women than in F samples. Gene-associated DMRs were also identified that are either hypomethylated (*n* = 54) or hypermethylated (*n* = 539) in both F and MyoF from Black women (Figure 4, C and D). We next determined which CpG probes showed the highest concordance in hypo- and hypermethylation between Black and White MyoF samples and all F and MyoF samples (Figure 4, E and F, respectively) to identify genes associated with differential methylation that correlated with race and could be important for the disparity in fibroid disease. We identified 327 gene-associated probes that were hypomethylated and 495 that were hypermethylated by greater than 10% in both F compared with MyoF and in Black compared with White MyoF.

**Figure 5:**
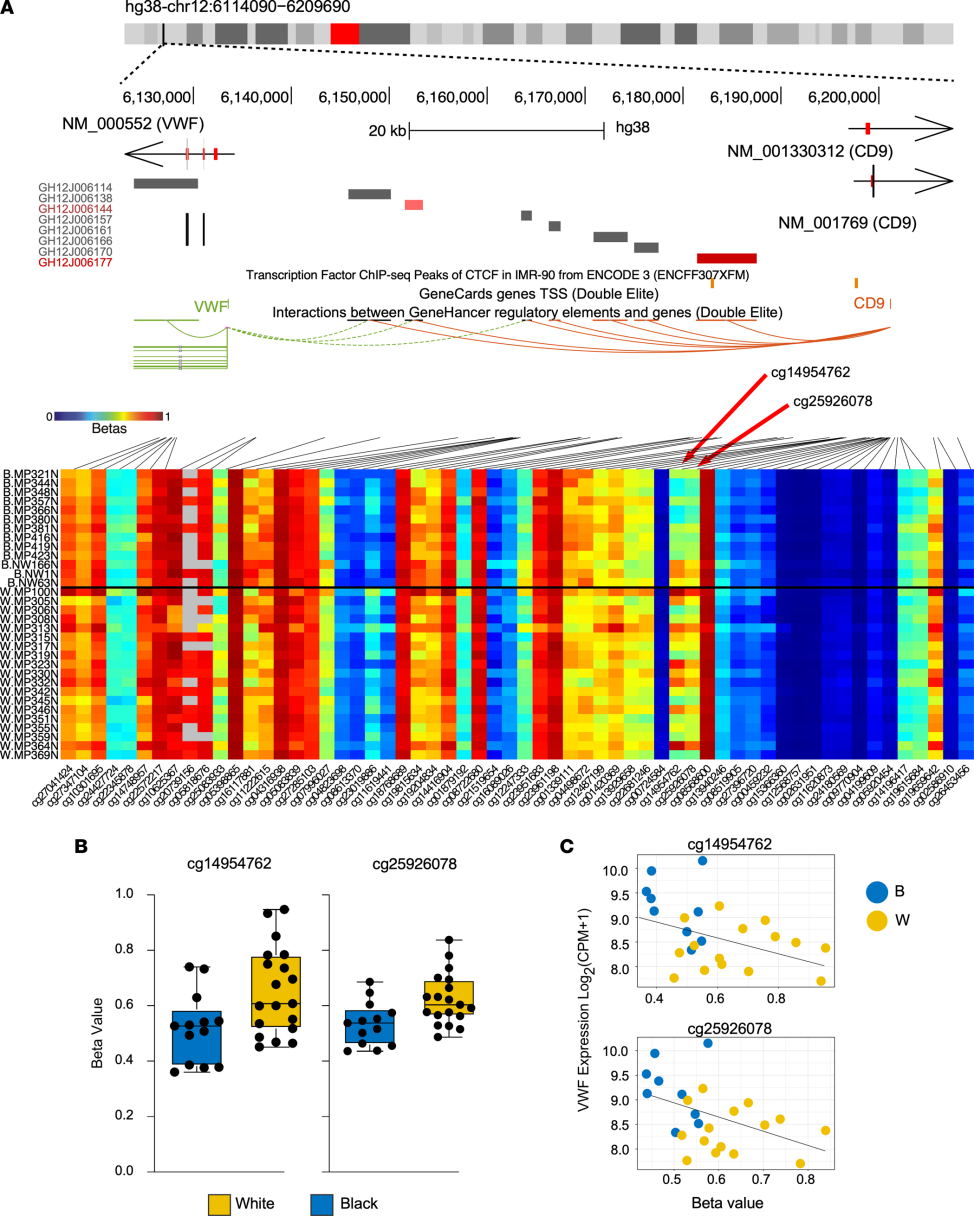
VWF gene hypomethylation correlates with increased gene expression. (**A**) UCSC genome browser view of the VWF gene locus, coupled with the DNA methylation β values for this region, shown as a heatmap (columns, CpGs; rows, samples grouped by race). Top UCSC tracks include locations CTCF ChIP-Seq peaks in IMR-90 cells from ENCODE (in orange) and predicted *cis*-regulatory elements provided by GeneHancer. The heatmap shows the CpG methylation in the VWF gene. (**B**) Box and whisker plots show the median and range of β values in Black and White MyoF for the 2 probes identified in **A**. Means for probes are significantly different by 2-tailed *t* test (*P* = 0.01 for both). (**C**) β Values (*x* axis) and VWF expression (*y* axis) show that β value is negatively correlated with expression in the MyoF samples (*r*^2^ = 0.19 for cg14954762 and *r*^2^ = 0.21 for cg25926078; *P* = 0.03 for both).

We observed 2 CpG probes associated with the *VWF* gene that were hypomethylated in both Black MyoF compared with White MyoF (Figure 5) and in F compared with MyoF (Supplemental Figure 2A). These hypomethylated probes, at approximately 60 kb upstream of the *VWF* transcription start site near a CTCF binding site (Figure 5A), identified by ChIP-Seq in IMR-90 smooth muscle cells (43), that might be important for regulatory interactions with putative enhancer elements (44). The probes are also upstream of another gene on the opposite strand, *CD9*, which is similarly upregulated in Black MyoF compared with White MyoF samples (Supplemental Figure 2B), suggesting coordinated regulation. One of these probes, which is hypomethylated in Black MyoF and in all F by more than the 10% cutoff, is indicated in Figure 4E in blue. The mean β values of both probes are significantly lower in Black MyoF than in White MyoF (Figure 5B). We also confirmed that the β value of each probe is consistent with *VWF* expression in the RNA-Seq results in each of the corresponding patient MyoF samples (Figure 5C).

## Discussion

There is a well-known racial disparity in uterine fibroid disease incidence and severity, in which Black women are more likely to have fibroids and have more and larger fibroids at diagnosis (45). In addition, Black women have higher rates of surgery for uterine fibroids than women of other races (46, 47). Previous studies investigating this disparity using transcriptomic or DNA methylation have been reported, but results did not show correlation by race with either gene expression (48) or DNA methylation (28, 49). By studying the race difference in both the fibroid tissue and the adjacent myometrium, we have discovered transcriptomic and DNA methylation differences between Black and White women that could be driving or contributing to the racial disparity in the disease.

We observed that, while RNA-Seq myometrial cluster 1 had a statistically significantly higher proportion of Black women and cluster 2 had a higher proportion of White women, the clusters were not strictly defined by patient race (Figure 1B). This suggests that shared exposures or experiences among cluster members could explain the differences between the clusters and can provide insight into fibroid formation. Although in the current study, women had 1–3 fibroids evenly distributed by race, Black women are known to be at higher risk to develop multiple and larger fibroids than White women (45), and a field effect by the fibroids on the surrounding myometrium could influence the transcriptomic and epigenetic profiles of the tissue. This caveat could confound our interpretation of the results and represent a limitation of this study. Expansion of the study by collection of and comparison of myometrial samples from 3 different cohorts of Black women — (a) without fibroids, (b) with only 1 fibroid, and (c) with multiple fibroids as confirmed by pathology — could be dispositive.

We have highlighted the differential expression of 3 genes (*BDNF*, *CCND1*, and *VWF*) in our results that we think could play key roles in uterine fibroid etiology or pathogenesis. *CCND1*, a cell cycle regulator that promotes cellular proliferation and is frequently overexpressed in human cancers (50), was previously identified by us (27, 28) and others (51–53) as a central gene whose expression is upregulated in F compared with both MyoF and MyoN. *CCND1* expression was not significantly dysregulated in MyoF from Black women compared with that from White women, despite the fact that its expression was increased 1.5-fold. We hypothesize that the increased expression of *CCND1* in myometria of Black women is still biologically important in the fibroid disparity, given the trend for increasing expression in F > MyoF > MyoN, and we speculate that our study was underpowered to detect a significant difference in the comparison of myometria from Black and White women. Serum levels of VWF are known to be elevated in Black individuals (29, 30), which provides us with a degree of confidence that these results are biologically meaningful. VWF, an endothelial and tumor progression marker (54), was upregulated in MyoF samples compared with myometrium from women without fibroids, but it was not differentially expressed in the F tissues (27). This result suggests that vascularization in MyoF from Black women could be higher than in MyoF from White women and could be similar in F from both, after the tumors are established. Indeed, microvessel density in F has been reported to be lower than in adjacent MyoF (31).

We showed that *BDNF* expression is higher in Black MyoF and in both F compared with White MyoF samples, the significance of which is not clear. *BDNF* has been associated with psychological stress (32), and serum BDNF appears elevated after acute psychosocial stress (33). However, another study has reported a conflicting correlation between *BDNF* expression and stress (55), which adds a degree of uncertainty to attributing a possible role to BDNF in fibroid biology. Black women are known to suffer more from socioeconomic stress than other races (56), and Black individuals experience more factors that negatively impact their health (e.g., perceived personal discrimination, perceived structural racism). Through weathering (the accumulation of negative effects) and John Henryism (the cost of coping with these insults), their individual health is further eroded. Additionally, community level factors (e.g., toxicants) both directly impact health status and modify the relationship between individual-level factors and individual health status (57, 58), suggesting that stress-associated genes could play a role in the establishment of uterine fibroids, particularly in Black women.

Interestingly, 3 genes, *ARL2*, *AIP*, and *CMYA5*, from a total of 33 in a peptide ancestry informative markers study of Black and White F tissues (59) were found in our DEG list from the Black MyoF comparison with White MyoF. The pathological significance of these genes to fibroid biology is not known, and further analyses are needed. The *AIP* gene is particularly interesting, since it encodes for the aryl hydrocarbon receptor-interacting protein (AIP) — mutation of which has been associated with pituitary adenoma development and acromegaly (reviewed in refs. 60, 61). It is possible that higher *AIP* expression in Black myometrial samples could be associated with higher levels of exposure to pollutants, and it is tempting to speculate that it provides the missing link between the higher incidence of fibroids in Black women with living in an urban environment. More studies are needed to explore this possible link in the general context of the role that urban environmental pollutant exposures have on fibroid development and growth.

Psychological stress and depression have also been shown to induce remodeling of the chromatin landscape, which affects gene expression (62, 63). Additionally, transgenerational epigenetic reprogramming has been associated with in utero and parental stress (reviewed in ref. 64), suggesting a repeating or even perpetual mechanism for maintaining health disparities in a variety of disease states in marginalized or oppressed communities. In our study, we showed that MyoF samples from Black women compared with those from White women have a similar number of hypomethylated gene-associated DMRs as in the F comparison. In contrast, more hypermethylated gene-associated DMRs were observed in the MyoF samples from Black women compared to those from White women than in F. In both cases, the number of overlapping DMRs was relatively small. Further study is needed to determine the biological importance of these striking and contrasting results.

Although *CCND1* and *BDNF* gene regions were not differentially methylated, 2 probes upstream of *VWF* were hypomethylated in MyoF from Black women compared with MyoF from White women, and in F samples compared with MyoF, results that were correlated with RNA-Seq results. Moreover, GSEA of the MyoF samples showed pathways known to be upregulated during stress — e.g., reactive oxygen species (reviewed in ref. 65) and hypoxia (66, 67) — and in uterine fibroid — e.g., Wnt/β-catenin signaling (68), TGF-β signaling (27, 69), and myogenesis (70). The results of this study provide a compelling rationale to include adjacent myometrial tissues in addition to the fibroid tissues in studies designed to better understand the etiology of fibroids and the racial disparity in the incidence of the disease. These data also suggest that the relationships between various exposures and epigenetic patterns in the fibroids and other uterine tissue could provide important information in fibroid etiology.

## Methods

### Tissue collection.

F samples and matched MyoF from White and Black premenopausal women (aged 37–52) were obtained following total hysterectomy. All women who participated in the study gave consent to donate tissue for this study through the Spectrum Health Biorepository. Human samples were processed as previously described (27). Briefly, samples were washed with phosphate-buffered saline, minced, and immersed in RNAlater (MilliporeSigma) and stored at 4°C for RNA-Seq analyses. The remaining tissue pieces were immediately flash frozen and stored at −80°C for methylation analysis. *MED12* mutation in the fibroids was determined by PCR amplification, followed by Sanger sequencing, as described in our previous study (27).

### RNA isolation.

Total RNA was isolated from frozen tissues stored in RNAlater. Tissues were homogenized in TRIzol reagent (Thermo Fisher Scientific), and RNA was isolated following the manufacturer’s instructions. Supplemental Table 6 contains all the metadata available for these samples. Isolated RNA was stored at −80°C in nuclease-free water. Nanodrop 1000 spectrophotometer (Thermo Fisher Scientific) and Agilent 2100 Bioanalyzer (Agilent Technologies) instruments were used to measure RNA concentration and quality, according to the manufacturers’ protocols. RNA integrity values of ≥ 7.5 were required for sequencing.

### Library preparation, and sequencing.

High-quality RNA samples from White F, Black F, White MyoF, and Black MyoF were submitted to the Van Andel Research Institute (VARI) Genomics Core for library preparation and paired-end (2 × 100 bp) RNA-Seq on an Illumina NextSeq 6000 instrument (Illumina). Libraries were prepared using a Kapa RNA HyperPrep kit with ribosomal reduction, pooled, and sequenced on flowcells to yield approximately 50–60 million reads/sample. Raw fastq files were deposited in the NCBI Gene Expression Omnibus (GSE207350).

### RNA-Seq analysis.

New reads and reads from samples in previously published studies (27, 28, 71) — Black F and matching MyoF (GSE128229), Black and White F and MyoF (GSE135446), and MyoN (GSE169255) — were trimmed for quality and adapters using TrimGalore (version 0.6.5), and quality trimmed reads were assessed with FastQC (version 0.11.7). Trimmed reads were mapped to *Homo sapiens* genome assembly GRCh38 (hg38) using STAR (version 2.7.9a) (72). Reads overlapping Ensembl annotations (version 99) were quantified with STAR prior to model-based differential expression analysis using the edgeR-robust method. Genes with low counts per million (CPM) were removed using the filterByExpr function from edgeR (72). Consensus clustering plots were made using the median-centered, normalized counts for the 5,000 most variable genes based on median absolute deviation and the ConsensusClusterPlus package (version 1.56.0) (73) with the parameters “reps=1000”, “pItem=0.8”, “pFeature=0.8”, and “distance=spearman”. Briefly, these settings resample the data 1,000 times using 80% of the samples and features each time; they then find the consensus clustering based on hierarchical clustering of each resampling using (1-Spearman correlation) as distance. Scatterplots of 2 selected PCs were constructed using the PCAtools package in R (version 2.5.13) to verify sample separation prior to statistical testing. Generalized linear models were used to determine if PCs were significantly associated with race by tissue. Genes were considered differentially expressed if their respective edgeR-robust FDR-corrected *P* values were less than 0.05. Differential expression was calculated by comparing Black F with White F, Black MyoF with White MyoF, or F with MyoF. DEGs were visualized with volcano plots and heatmaps generated using the EnhancedVolcano (version 1.6.0) and pheatmap (version 1.0.12) packages, respectively, in R. Downstream analyses of RNA-Seq results were completed using the clusterProfiler (version 3.16.1) (74) package in R with an FDR *P* value cutoff of 0.05. GSEA were conducted with all expressed genes using the 50 Hallmark gene sets collection (34) downloaded from the Molecular Signatures Database (MSigDB) (35). The top enriched GSEA terms were shown in the figures. Venn diagrams were constructed to visualize overlapping genes between groups or gene sets using the eulerr package (version 6.1.1). DOSE R package (version 3.14.0) was used to generate the leiomyoma gene set heatmap using DOID:127 (75). Box plots of the log_2_(CPM + 1) values were generated using the R package ggplot2 (version 3.3.5). Independent MyoF samples (*n* = 4 MyoF White and *n* = 4 MyoF Black) from another leiomyomas study (40) were used to confirm our RNA-Seq analysis (GSE193320).

### DNA isolation and DNA methylation analysis.

DNA was isolated from snap-frozen myometrium and fibroid tissue samples (Supplemental Table 4), and it was hybridized to the Infinium MethylationEPIC array and analyzed essentially as previously described (28). Raw IDAT files were deposited in NCBI GEO (GSE207350). Additional raw IDAT files from our previous report (28) GSE120854 and GSE135446 were added. Briefly, raw IDAT files were processed using R package SeSAMe (version 1.12.7) and the openSesame pipeline with noob background correction, nonlinear dye bias correction, and nondetection masking (42, 76). DNA methylation was measured in β values for each probe calculated as a quantitative percentage of methylated signals over both methylated and unmethylated probe signals. Matched samples and self-identified race were confirmed using EPIC SNP probes (Supplemental Figure 3, A and B) with a published model (42), and cellular composition of the samples was determined by promoter methylation of *MIR200C/141* (77) and *ACTA2* (78) (Supplemental Figure 3C).

MDS analysis of the top 500 most variable CpG probes was performed using R package minfi (version 1.40.0). DMRs were called using R package SeSAMe by first modeling the variation in DNA methylation in each CpG probe using race as the independent variable. Neighboring CpG probes that showed consistent variation in methylation were then merged into DMRs. Visualization of DNA methylation in probes across a genomics region was generated using University of California Santa Cruz (UCSC) genome browser tracks from available track hubs including GeneHancer regulatory elements (44) and gene interactions and CTCF ChIP-Seq peaks in IMR-90 (43). Probe annotation of Illumina EPIC array (human reference genome; NCBI build GRCh38/hg38) was downloaded from https://www.ncbi.nlm.nih.gov/pmc/articles/PMC5389466/bin/gkw967_supplementary_data.zip (42).

### Statistics.

Bioinformatic statistics were performed using the listed packages in R (version 4.0.2). DEGs were identified as those having an Benjamini-Hochberg FDR–corrected *P* < 0.05 (79). Data with unequal variances were log transformed, and homogeneity of variances was verified before completion of analyses. Hypergeometric testing was performed using the phyper function from the stats package (version 4.0.2). and the Likelihood ratio tests were done with lrtest function using the lmtest package (version 0.9-40). Comparison of 2 means was performed with a 2-sided Student’s *t* test, and significance was determined at *P* < 0.05 after confirming normal distribution using Graphpad Prism (version 9.3.1). CpG β correlations with expression were also performed with Prism.

### Study approval.

The use of human tissue specimens was approved by the Spectrum Health Systems and Michigan State University IRBs (MSU IRB Study ID STUDY00003101, Spectrum Research IRB 2017-198) as secondary use of biobank materials.

## Author contributions

Experimental design was contributed by ENP, RLC, HS, and JMT. ENP, JAG, and TJC collected data and performed experiments. ENP, JAG, TJC, ZBM, KHL, SAG, and JMT analyzed data. ENP, JAG, TJC, ZBM, KHL, SAG, GWB, RLC, GRW, HS, and JMT wrote and reviewed manuscript.

## Supplementary Material

Supplemental data

Supplemental table 1

Supplemental table 2

Supplemental table 3

Supplemental table 4

Supplemental table 5

Supplemental table 6

## Figures and Tables

**Table 1:**
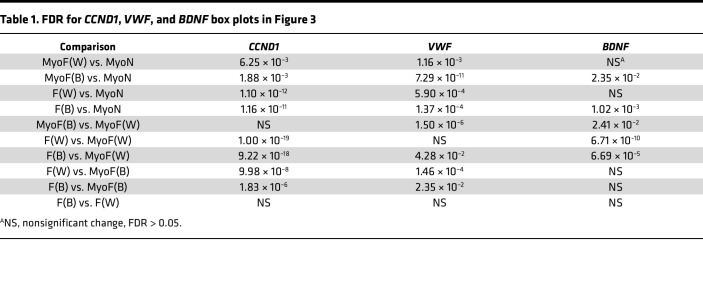
FDR for *CCND1*, *VWF*, and *BDNF* box plots in Figure 3

## Version 1 09/06/2022

In-Press Preview

## Version 2 10/24/2022

Electronic publication
