# Supplementary material for: Transcriptome and DNA methylome analyses reveal underlying mechanisms for the racial disparity in uterine fibroids
Source: JCI Insight. 2022 Oct 24;7(20):e160274. doi: 10.1172/jci.insight.160274 (PMC9714787; doi:10.1172/jci.insight.160274)
Supplement: Supplemental data [file jciinsight-7-160274-s007.pdf]

Figure S1

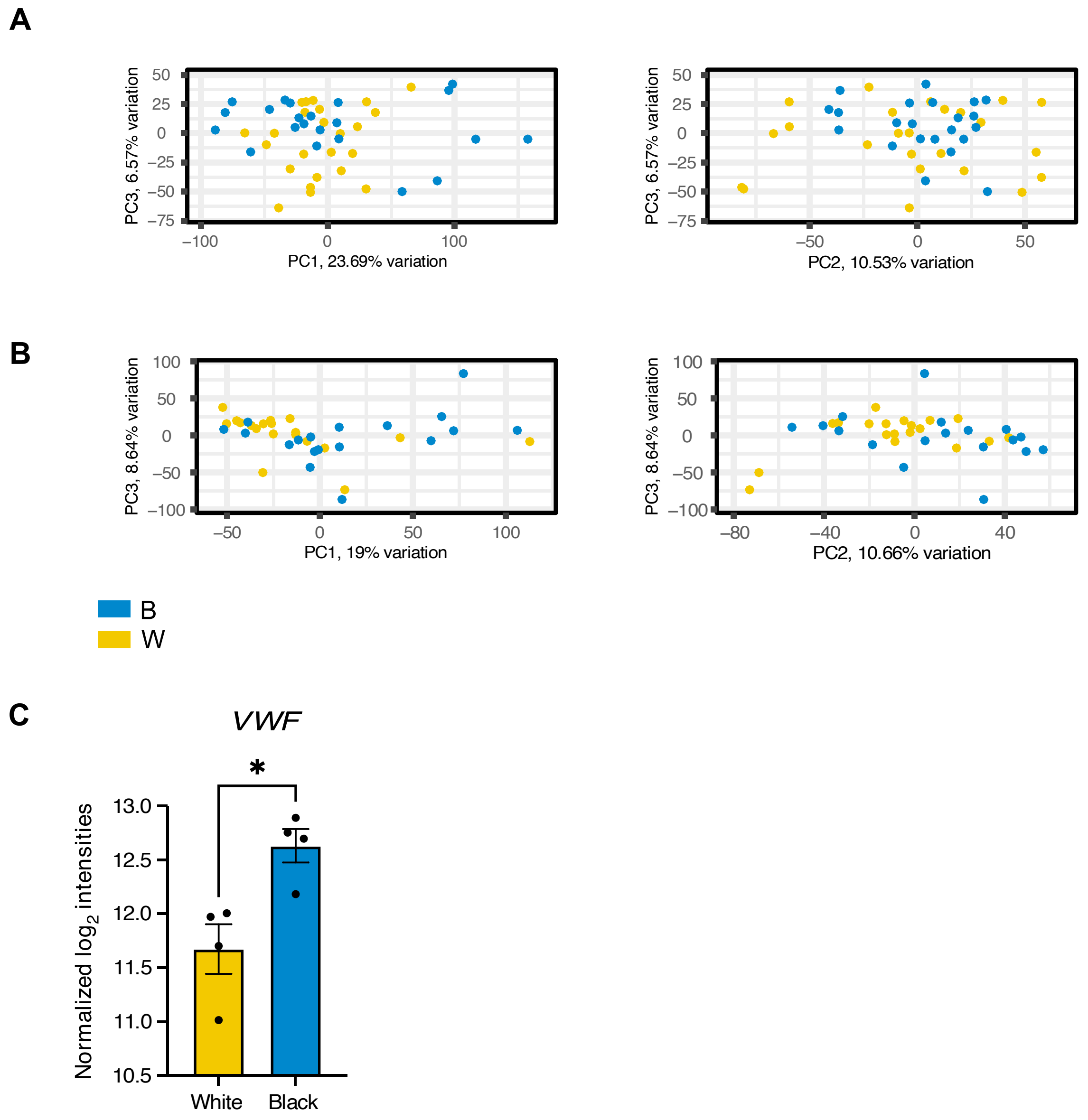

Figure S1. Principal component analyses of RNA-seq results from Fibroids (**A**) and Myometrial (**B**) samples from Black and White women. (**C**) Microarray results (GSE193320; Chuang, T.D., et al., *Differential Expression of Super-Enhancer-Associated Long Non-coding RNAs in Uterine Leiomyomas.* Reprod Sci, 2022.) for *VWF* expression in myometrial samples from Black and White women. *p<0.05 by two-tailed student t test.

Figure S2

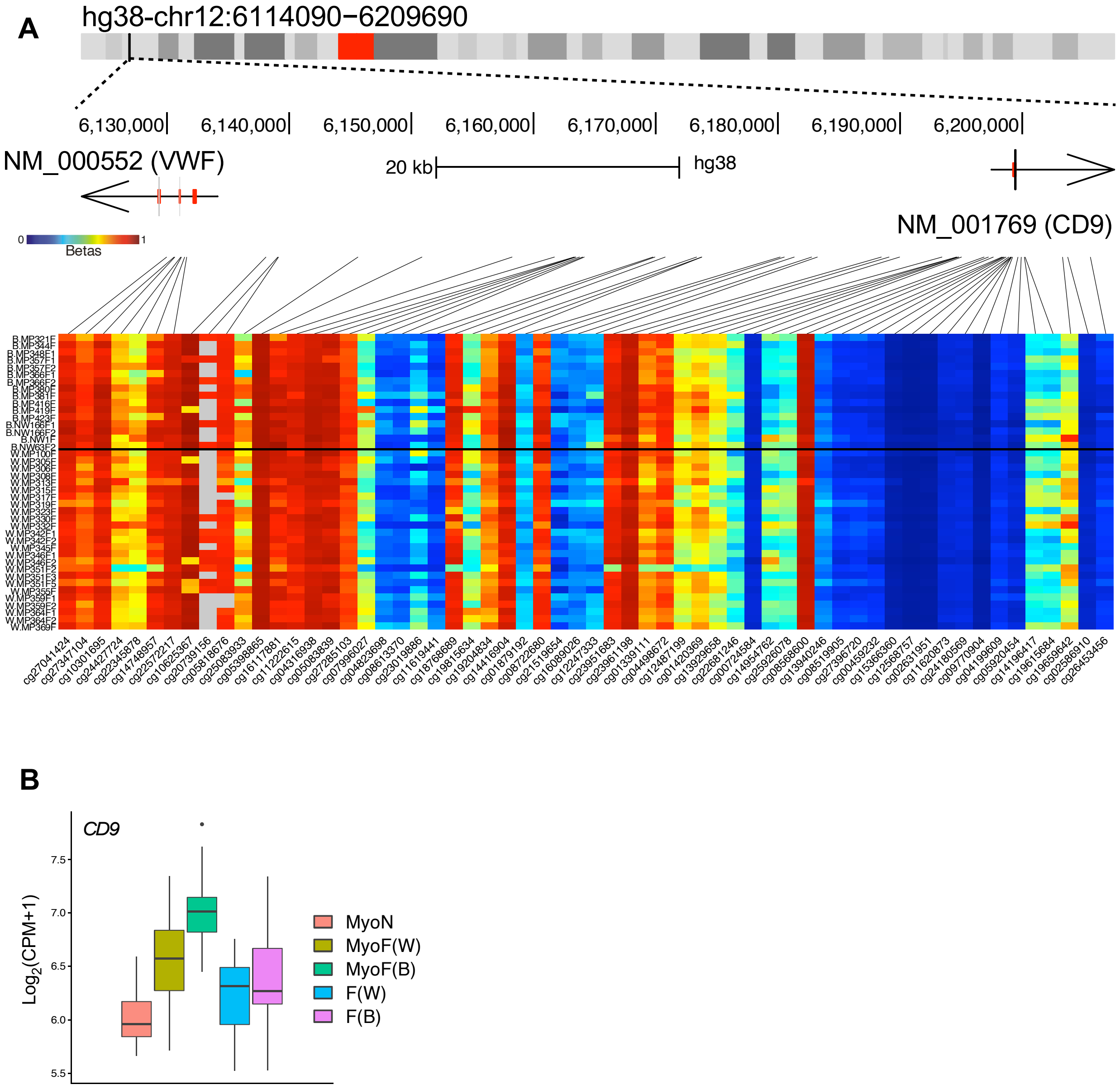

Figure S2. DNA methylation at the VWF and CD9 genes. **(A)** Heatmap of the CpG probes methylation beta values in fibroids. The white box indicates the position of the 2 CpG probes that are differentially methylated in myometrial samples (Fig 5). The black line separates fibroid samples from Black and White women.
(B) Box and wisker plot for CD9 expression.

Figure S3

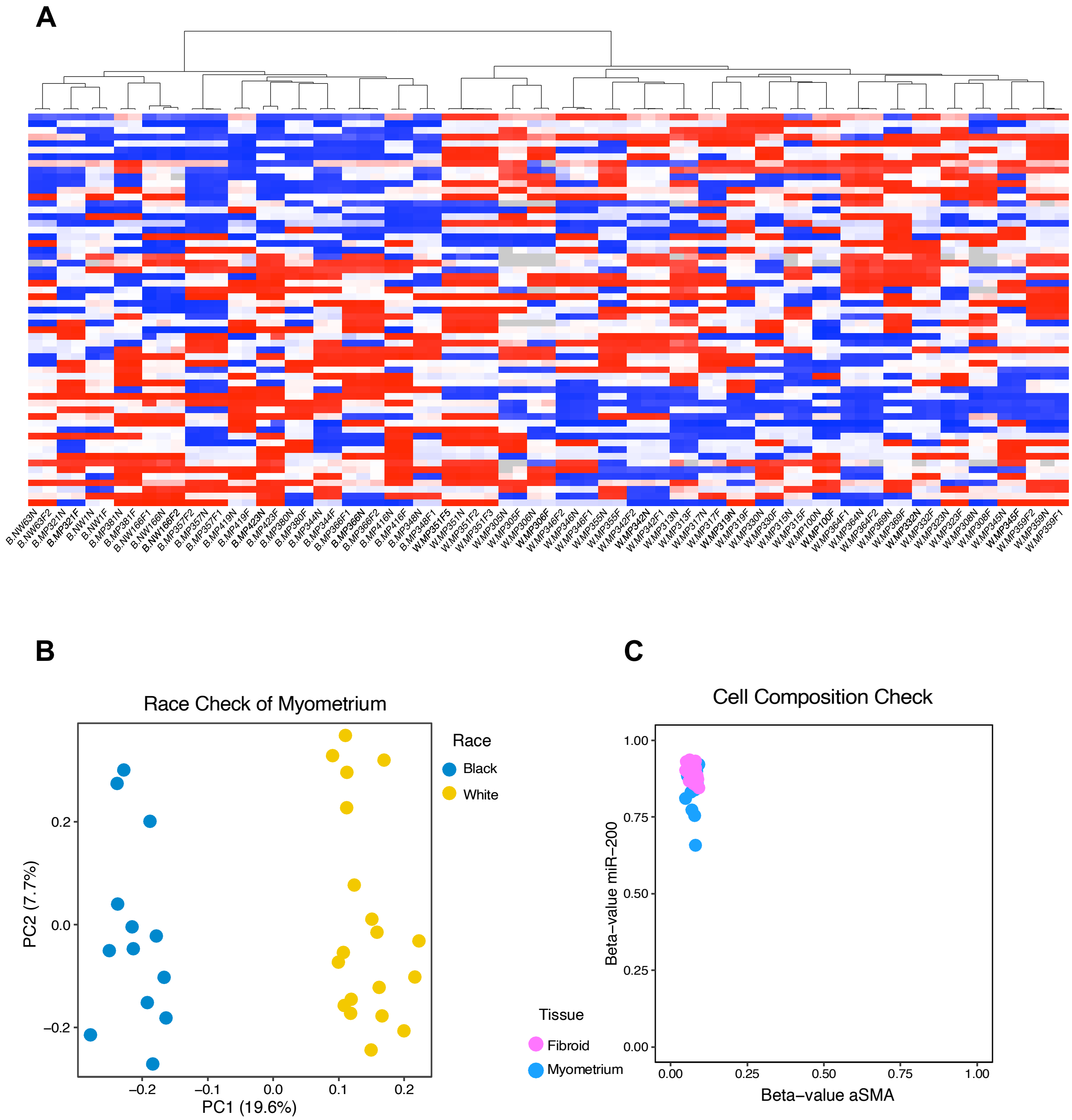

Figure S3. Sample quality control. (A) SNPs were determined from the EPIC Array data and used to generate a heatmap in tissues. Samples from the same patient a found in a single branch. (B) Principal component analysis of race-specific SNPs were used to ensure that only samples with congruent self-reported race and SNPs were subsequently analyzed. (C) Cell composition by beta values for aSMA (smooth muscle-specific gene) and miR-200 (epithelial-specific).
